# Unveiling Complexity: Mathematical Models in Aortic Disease Investigation

**DOI:** 10.3390/bioengineering11121260

**Published:** 2024-12-12

**Authors:** Hwa Liang Leo, Hamed Keramati

**Affiliations:** 1 Department of Biomedical Engineering, National University of Singapore, Singapore 117583, Singapore; bielhl@nus.edu.sg; 2 Department of Biomedical Engineering and Imaging Sciences, King’s College London, London SE1 7EH, UK

The complexity of aortic diseases demands sophisticated modeling approaches to better understand their pathophysiology and optimize treatment strategies. As our understanding of cardiovascular diseases continues to evolve, it has become increasingly apparent that the interaction between biomechanics, hemodynamics, and tissue biology plays a crucial role in both disease progression and treatment outcomes. Mathematical and computational modeling have emerged as powerful tools in this context, offering insights that would be impossible to obtain through traditional clinical observations alone [1].

The landscape of aortic diseases encompasses a wide spectrum of pathologies, from valvular conditions, such as aortic stenosis and bicuspid aortic valve disease, to aortic wall pathologies, including aneurysms and dissections. Each of these conditions presents unique challenges in terms of diagnosis, risk stratification, and treatment planning. Traditional clinical approaches, while valuable, often rely on simplified metrics that may not capture the full complexity of the underlying disease processes. This is particularly evident in cases where multiple pathophysiological mechanisms interact, such as the combination of valvular disease with altered aortic wall properties, or when considering the impact of flow patterns on thrombosis risk.

The rapid advancement of computational capabilities and imaging technologies is ushering in an era where sophisticated mathematical modeling can be integrated into clinical practice in meaningful ways. High-performance computing allows for increasingly complex simulations, while medical imaging improvements provide more detailed anatomical and functional data to inform these models. Computational advancement has enabled us to isolate pathological factors and investigate aortic diseases systematically [2] or perform in silico sensitivity analyses [3], which provide invaluable insights to guide clinical research and trials. The convergence of these technical and technological advances creates unprecedented opportunities for developing more accurate and clinically relevant modeling approaches.

This Special Issue presents five innovative studies that demonstrate the breadth and depth of mathematical and computational modeling and their potential in advancing our understanding of aortic diseases and their treatments. The selected works represent various aspects of contemporary cardiovascular modeling, from detailed fluid–structure interaction analyses to novel physiologically based algorithms. They showcase how mathematical modeling can bridge the gap between basic science and clinical application, providing insights that can directly impact patient care.

Moreover, the growing adoption of minimally invasive techniques, such as transcatheter aortic valve replacement (TAVR) and endovascular repair of aortic pathologies, has created new challenges in predicting and optimizing procedural outcomes. These challenges can be effectively addressed through computational modeling, as demonstrated by several studies in this Issue. The ability to simulate and predict the behavior of devices and their interaction with patient-specific anatomy before intervention makes it a powerful tool for procedural planning and risk assessment.

Kovarovic et al. demonstrate this through their innovative analysis of paravalvular leak in transcatheter aortic valve replacement (TAVR) [4]. Their study combines in vitro patient-specific TAVR replicas with sophisticated computational fluid dynamics to examine flow patterns and platelet trajectories through paravalvular leak channels. By utilizing high-resolution μCT scans to reconstruct in silico models, they reveal how even mild leaks classified as clinically acceptable may pose increased thrombogenic risks in specific patient anatomies. This work challenges current clinical paradigms that often rely on simplified classifications of paravalvular leak severity and highlights the need for more nuanced, patient-specific evaluation methods.

Similarly, Sophocleous et al. establish the feasibility of deriving wave intensity analyses from 4D cardiovascular magnetic resonance imaging data in patients with bicuspid aortic valve disease [5]. Their methodology represents a significant advance in our ability to study arterial wave dynamics non-invasively. By analyzing data from multiple planes along the aorta, they demonstrate the possibility of studying wave travel and reflection patterns specific to individual patients. This patient-specific approach to wave intensity analysis could lead to an improved understanding of vascular function in various pathological conditions and better-tailored therapeutic strategies.

The importance of order reduction for patient-specific aortic disease modeling is demonstrated by Çelikbudak Orhon et al.’s work on aortic stenosis evaluation [6]. Their 1D mathematical model incorporates individual patient parameters of left ventricular function and arterial load, demonstrating how these factors significantly influence the assessment of aortic stenosis severity. This approach moves beyond traditional pressure gradient measurements to provide a more comprehensive evaluation of the disease process in each patient.

The evolution of patient-specific modeling is also evident in Alzhanov et al.’s development of a physiologically based algorithm for coronary flow assessment [7]. Their work demonstrates how patient-specific boundary conditions and anatomical features can be incorporated into computational models to provide more accurate predictions of coronary flow dynamics. This represents a significant step toward more accurate personalized diagnostic tools that could potentially reduce the need for invasive procedures while maintaining diagnostic accuracy.

Sengupta et al.’s analysis of endograft performance in complex aortic arch repair further emphasizes the importance of patient-specific considerations in treatment planning [8]. Their computational modeling approach accounts for individual variations in aortic arch anatomy and flow patterns, providing insights into how these factors influence treatment outcomes. This work particularly highlights how aortic flow modeling can aid in the selection and optimization of medical devices for individual patients.

These advances in aortic disease modeling are made possible by improvements in imaging technology, computational capabilities, and our understanding of cardiovascular physiology [9,10]. The integration of multiple data sources—including anatomical imaging, flow measurements, and clinical parameters—allows for increasingly sophisticated and accurate models. However, these studies also highlight the challenges in implementing such detailed analyses in clinical practice, including the need for standardization of modeling approaches, validation of results, and development of more efficient computational methods.

The studies presented in this Special Issue highlight several critical paths forward for the field of aortic disease modeling. Validation and verification of computational models remain fundamental challenges that must be addressed through systematic comparison with clinical data and standardized protocols. While current models show promising results, establishing their reliability across diverse patient populations and clinical scenarios is essential for widespread adoption in medical practice.

The integration of multiple physiological parameters in disease assessment represents another crucial direction for future development. As demonstrated by several studies in this issue, comprehensive modeling approaches that consider both cardiac and vascular function provide more accurate insights into disease progression and treatment outcomes. Future work should focus on developing multi-scale models that can efficiently integrate molecular, cellular, and organ-level processes while maintaining clinical practicality.

As we move forward, the focus should be on developing solutions that can be readily implemented in clinical practice while maintaining the sophisticated analysis capabilities demonstrated in these studies. The works presented in this Special Issue provide a strong foundation for these future developments while highlighting the potential of mathematical modeling to significantly advance cardiovascular medicine.
